# Aducanumab for Alzheimer’s disease: The never‐ending story that nurses should know

**DOI:** 10.1002/nop2.878

**Published:** 2021-04-07

**Authors:** Joel Petch, Daniel Bressington

**Affiliations:** ^1^ Kent and Medway Medical School Canterbury UK; ^2^ College of Nursing & Midwifery Charles Darwin University Casuarina NT Australia

In October 2019, the American pharmaceutical company Biogen stated intention to apply to the US Food and Drug Administration (FDA) for the candidate drug aducanumab to be licenced as a therapeutic agent for the treatment of Alzheimer's disease (AD). If approved, aducanumab would become the first new medication licenced specifically for the treatment of AD in the past decade; and approval in the US may be a precipitant for approval elsewhere.

Predictably, the potential advent of a new drug for AD has resulted in considerable excitement amongst the general population, healthcare professionals and AD charities alike (Alzheimer’s Research UK, 2019). This enthusiasm is understandable as aducanumab has been posited as a medication that can reduce brain amyloid accumulations and reduce clinical decline seen in AD (Biogen, 2019a; Wang & Holtzman, 2020), which is currently viewed as a progressive and irreversible neurodegenerative condition with cerebral atrophy, cognitive decline and a loss of functionality for the individual (Wang & Holtzman, 2020).

Currently approved evidence‐based pharmacological agents for AD include acetylcholinesterase inhibitors, such as donepezil and galantamine, and the glutamatergic antagonist memantine (BNF, 2021). An important distinction to make is that presently approved medications are considered to slow the progression of AD whereas aducanumab has been stated to reverse the pathological process, and associated clinical decline, by way of clearance of extracellular amyloid beta plaques observed within AD (Carroll, 2020). If aducanumab really can reverse these features and reduce the cognitive decline, this would be a revolutionary treatment.

Whilst on the surface this sounds a very positive advance with significant potential, the decision by Biogen to pursue FDA approval has raised some concerns (Carroll, 2020). Aducanumab – a high‐affinity, fully human IgG1 monoclonal antibody – is actually not a new drug; it was first produced by *Neurimmune* and its licence was sold to Biogen in 2007 (Neurimmune, 2020). There have been several earlier studies exploring aducanumab within the treatment of AD which failed to demonstrate efficacy when compared to placebo. This apparent lack of response to aducanumab within clinical trials had led to Biogen's own phase three trials being discontinued in early 2019 (Biogen, 2019a). Despite discontinuing the trials, Biogen undertook a retrospective data trawl which has appeared to uncover incorrect analysis earlier in the study, leading Biogen to claim that the original trial dose was too small and that one of the two‐phase three clinical trials [*the EMERGE trial*] actually did reduce clinical decline in patients with early AD (Biogen, 2019b). This statement of success appeared conflicted with an earlier disclosure highlighting that the trial did not need the pre‐specified end point, so confusion abounds. This new analysis of the data was reported to have observed a “*statistically significant reduction in clinical decline in early AD patient*s,” and it has been claimed that greater exposure to high dose aducanumab was the primary driver for the apparent positive results (Biogen, 2019a).

Following Biogen's announcement to seek FDA approval which was declared without full disclosure of supporting data at the time‐independent analyses was undertaken following public presentation of the data during on 5th December 2020. These data have split opinion with regard to the potential efficacy for AD with protocol amendments, initiated mid‐study in 2017, proving problematic for many reviewing the data. The conflict as to whether the data presented were a true reflection of an objective and transparent trial remains palpable. Indeed, the UK Alzheimer’s Society (2019; Alzheimer’s Research UK, 2019) state it is not possible to determine whether aducanumab is effective, yet the US Alzheimer's Association are currently strongly advocating for approval by the FDA (Alzheimer’s Association, 2020); even stating that a further delay to undertake additional clarifying trials would not be acceptable. AD charities have an influence about the hopes of people living with dementia, their carers and many healthcare professionals; and it could be argued that advocating for FDA approval may be providing a higher level of optimism than is perhaps realistic. Given the important role of many charitable organisations, it is of paramount importance that the public are aware of the conflicting messages emanating from what are two of the most well‐known AD charities, to permit objective discussions based on the evidence.

Currently, we are still awaiting a decision by FDA as to whether approve aducanumab for clinical practice. This decision was due to be made in March 2021 but has now been delayed until 7 June 2021. This delay is following an FDA request for additional data from Biogen, which has been provided. This should be viewed in the context of an FDA advisory committee which met in November 2020 and raised significant concerns questioning the effectiveness of aducanumab. The outcome of this advisory committee appeared to suggest that the application would be rejected, with the panel voting against approving aducanumab. Despite this, it is noteworthy that the FDA is not obliged to follow the committee's advice (Bowman Rogers, 2020). Interestingly, the peaks and troughs of optimism about this candidate drug have been reflected in Biogen's stock market status.

On a wider perspective, few could argue that it is not exciting that there is renewed scientific and pharmaceutical interest within the area of AD, something which appears to have reduced over recent years, perhaps due to the complexity of AD pathophysiology. Aducanumab may indeed turn out to be the first medication which removes extracellular accumulations of amyloid beta, but others may follow. These include both donanemab and gantenerumab, amongst others, and there is renewed life within these candidate drugs following on from the apparent positive findings related to aducanumab (Adams, 2021). However, the inescapable reality is that AD pathophysiology is highly complex. This is demonstrated further still when considering that the accumulation of amyloid beta, the target of aducanumab and other candidate drugs, continues to be poorly associated with levels of cognition and that clearance of amyloid does not appear to be commensurate with improvement to cognition (Ackley et al., 2021).

So, what might this mean for mental health care and nursing practice in 2021 and beyond? Well, much of this depends upon the FDA and their decision about approval; along with the decisions made by further regulatory organisations worldwide. At best, a further medication may be available, although as it would be based upon the current data set it may be prudent to scrutinise the data and to ask just how beneficial this may be. There are further considerations also – at a time when many economies have regressed because of the SARS‐CoV‐2 pandemic – of where and how future health spending is undertaken is critically important. Our current psychopharmacological interventions remain affordable options with a good evidence base for efficacy in mild‐moderate AD. These medications augmented with comprehensive, person‐centred psychosocial interventions and skilled nursing input must remain at the centre of therapeutic interventions. The approval of a costly new medication with potentially dubious effectiveness could jeopardise care packages which are evidenced to increase the quality of life for those with a diagnosis of AD.

It could be argued that this situation may illustrate a broader issue of how pharmaceutical companies can influence treatment hopes. Further, this circumstance also demonstrates how the recommendations of respected charities (which may well be the primary source of health literacy information for the general public), when based on preliminary findings that are not peer‐reviewed or subject to the usual checks and balances of the scientific publishing process, could have an adverse effect on the quality of life for those living with a diagnosis of dementia and their families.

In summary, the availability of new medications that can reduce accumulations of amyloid beta in AD is an exciting prospect. This potential treatment development is of great interest to all stakeholders and is likely to become a topic of discussion within clinical meetings with nurses that work with people who live with mild‐moderate AD and their carers. However, nurses need to know that clearance of amyloid does not necessary improve cognition in AD, that the evidence of effectiveness of aducanumab remains unclear (despite strong support from the US Alzheimer's Association) and that currently the best evidence‐based treatment approaches involve person‐centred psychosocial interventions combined with acetylcholinesterase inhibitors and memantine.

## CONFLICT OF INTEREST

Daniel Bressington is Associate Editor of Nursing Open. There are no other conflicts of interest for both authors.

## AUTHOR CONTRIBUTIONS

Both authors have contributed equally, meet at least one of the ICMJE criteria for authorship (http://www.icmje.org/recommendations/) and have approved the final version.

## ETHICAL APPROVAL

Ethical approval is not required for this editorial.

## Data Availability

Data sharing not applicable – no new data generated.
